# Optimizing dietary tryptophan in quail chicks: Implications for NRC (1994) recommendation

**DOI:** 10.1016/j.psj.2025.106136

**Published:** 2025-11-18

**Authors:** Mehran Mehri, Mahmoud Ghazaghi, Morteza Asghari-Moghadam, Amir Karamzadeh-Dehaghani, Mohammad Rokouei

**Affiliations:** a Department of Animal Sciences, Faculty of Agriculture, University of Zabol, Sistan 98613-35856, Iran; b Department of Animal Sciences, Campus of Agriculture and Natural Resources, University of Tehran, Karaj 77781-31587, Iran

**Keywords:** Dressing, Hepatic enzyme activity, Rational model, Tryptophan requirements

## Abstract

This study evaluated the effects of graded dietary tryptophan (Trp) supplementation on growth performance, carcass traits, hepatic enzyme activities, and immune response in growing Japanese quails. A total of 525 seven-day-old quail chicks were distributed in a completely randomized design with seven dietary Trp levels (0.21 %, 0.22 %, 0.23 %, 0.24 %, 0.25 %, 0.26 %, and 0.27 %) and five replicates of 15 birds each. Growth performance traits, including weight gain (G) and feed conversion ratio (FCR), responded significantly (*P* < 0.05) to increasing dietary Trp. The rational regression model estimated optimal Trp levels of 0.237 % for G and 0.238 % for FCR, corresponding to 7.9 % and 8.3 % higher requirements than NRC (1994) recommendations, respectively. Hepatic enzyme activities exhibited pronounced quadratic responses, with minimum alkaline phosphatase (ALP) and aspartate aminotransferase (AST) activities estimated at 0.242 % and 0.232 % Trp, representing 10.2 % and 5.6 % higher requirements than NRC (1994), respectively. The antibody titer against sheep red blood cells peaked at 0.247 % Trp (12.2 % higher than NRC, 1994), indicating enhanced immune responsiveness. Carcass traits, including thigh and breast meat yields, were optimized at 0.249 % and 0.251 % Trp, exceeding NRC recommendations by 13.0 % and 14.1 %, respectively. Overall, the estimated optimal Trp requirements for maximizing growth, immunity, and carcass yield ranged from 5.6 % to 14.1 % above NRC (1994) values, underscoring the elevated amino acid demands of modern quail genotypes. These findings highlight the need to update current nutritional guidelines to accommodate contemporary genetic potential, improved metabolic efficiency, and functional amino acid utilization, thereby supporting optimal growth performance, liver health, and immune competence.

## Introduction

Tryptophan (Trp) is an indispensable amino acid in poultry nutrition, serving as a precursor for several bioactive molecules that are critical for growth, metabolic homeostasis, and immune regulation in birds (Khanipour et al., 2019). In Japanese quail (*Coturnix coturnix japonica*), the requirement for Trp has traditionally been guided by values established by the NRC (1994), which recommended 0.22 % of diet for optimal growth and health. However, dietary Trp levels recommended by the NRC (1994) appear insufficient to meet the physiological demands of modern Japanese quail strains. El-Kholy et al. (2024) demonstrated that supplementing the basal NRC (1994)-based diet with 0.01 % Trp significantly enhanced growth performance, antioxidant status, and humoral immunity, indicating that higher Trp levels are required to optimize productivity and immune competence in contemporary quail genotypes. Mehri et al. (2021) and Ghazaghi et al. (2024), working with Japanese quails aged 21–35 days, demonstrated that the Trp requirements for optimal performance are substantially higher than those proposed by the NRC (1994). Mehri et al. (2021) reported that 0.28 % of diet was required to achieve maximum G, feed conversion ratio, and carcass yield, while Ghazaghi et al. (2024) found that around 0.27 % of diet optimized immune and physiological traits, including serum IgY, uric acid, triglycerides, and water-holding capacity. Collectively, these results indicate that for quails in this growth phase, the optimal Trp level is more than 21 % higher than the NRC (1994) recommendation, underscoring that modern strains have greater amino acid requirements to fully support both growth performance and physiological resilience.

The immunomodulatory and stress-buffering roles of Trp in quails are increasingly recognized, with higher dietary levels correlating with elevated lymphocyte counts, improved antibody production, reduced stress biomarkers, and even detoxification of aflatoxicosis (Khanipour et al., 2019; Linh et al., 2021). These effects are attributed to Trp’s involvement in synthesizing serotonin, melatonin, and kynurenine, which regulate neuroimmune interactions, antioxidant defense, and cellular stress responses (Fouad et al., 2021; Ghazaghi et al., 2024). Accordingly, higher Trp intake supports not only growth and carcass yield but also liver health and immune competence, with studies reporting significant improvements in hepatic enzyme activities and humoral immunity markers such as antibody titers against sheep red blood cells (Ghazaghi et al., 2024).

Despite growing evidence that modern quail genotypes have greater amino acid requirements than those proposed by the NRC (1994), there is still limited quantitative information describing the Trp needs of young quails during the early growth phase (7–21 days). Most previous studies have used simplified mean-based comparisons rather than nonlinear modeling to determine nutrient optima. Therefore, a clear, model-based estimation of Trp requirements for current Japanese quail strains remains lacking. The aim of this study was to determine the Trp requirements of young Japanese quail chicks during the first three weeks of age from 7 to 21 d of age, using graded dietary supplementation to evaluate impacts on growth, hepatic function, and immune response. The period from 7 to 21 days represents the most rapid growth phase in Japanese quails, characterized by high rates of protein accretion, rapid development of breast and thigh muscles, and maturation of hepatic and immune functions. This early growth window provides a physiologically relevant and experimentally stable period for determining dietary Trp requirements and provide an updated estimate of dietary Trp needs during the early growth phase, considering the elevated amino acid demands of modern quail genotypes using a nonlinear modeling approach.

## Materials and methods

### Ethics statement

This study protocol adheres to the guidelines established by the Iranian Council of Animal Care and it has been approved by the Research Animal Ethics Committee (AEUOZ-2012|UP-2020-BR) at the University of Zabol.

### Bird management

A total of 525 seven-day-old Japanese quail chicks (unsex chicks) with average weight of 24.5 ± 2.34 g were used in this study. The experiment was conducted in a completely randomized design consisting of seven dietary treatments with 5 replicates per treatment and 15 birds per replicate (100 cm length × 70 cm width × 40 cm height), making a total of 35 floor pens. The chicks were fed a standard NRC (1994) starter diet from hatch to 7 days of age and then randomly distributed into floor pens. The experimental house was maintained at 26 ± 2.3°C during the third week of age, with a relative humidity of 60 ± 3.4 %. The lighting schedule consisted of 16 hours of light (16 L) and 8 hours of darkness (8D) throughout the experimental period. Birds had *ad libitum* access to feed and water.

### Experimental Diets

The basal diet was balanced for all essential amino acids except Trp, using crystalline sources of lysine, methionine, threonine, valine, and isoleucine to meet or exceed NRC (1994) requirements. This ensured that Trp was the primary limiting amino acid and that responses were not confounded by deficiencies or imbalances of other amino acids. The basal diet was supplemented with graded levels of l-Trp at the expense of cornstarch to create seven experimental diets containing 0.21 %, 0.22 %, 0.23 %, 0.24 %, 0.25 %, 0.26 %, and 0.27 % Trp. All protein-containing ingredients and mixed experimental diets were analyzed for crude protein (method 990.03, AOAC, 2006) and amino acid composition (method 982.30, AOAC, 2006) prior to the start of the experiment. Amino acid analysis followed the procedure described by Hasanvand et al. (2018): feed samples were hydrolyzed for 24 h in 6 N HCl at 110°C under nitrogen; performic acid oxidation preceded hydrolysis for methionine and cystine determination; and Trp was analyzed after barium hydroxide hydrolysis. Amino acid separation was performed using a Waters HPLC system (Waters, Milford, MA).

### Growth performance measurements

Weight gain (G), feed intake (F), and feed conversion ratio (FCR) were measured on a pen basis from 7 to 21 days of age. Birds and feed were weighed at the beginning (7 d) and at the end (21 d) of the experimental period. BWG was calculated as the difference between final and initial body weights. FCR was calculated as the ratio of feed intake to G (g feed/g gain).

### Carcass traits

At 21 d of age, four birds per replicate were randomly selected and euthanized by cervical dislocation. Carcasses were defeathered and eviscerated, and the weights of breast and thigh muscles were recorded. Breast muscle yield (BMY) and thigh muscle yield (TMY) were expressed as a percentage of eviscerated carcass weight.

### Serum biochemical analysis

Immediately after slaughter (21 d of age), blood samples were collected from the jugular vein into 5 mL heparinized tubes. Samples were centrifuged at 1,500 × *g* for 5 min at 8°C, and the serum was separated and stored at −80°C until biochemical analyses. Serum aspartate transaminase (AST) and alkaline phosphatase (ALP) concentrations were determined using spectrophotometric methods and commercial diagnostic kits (Parsazmun, Tehran, Iran; AST kit catalog no. 89021, lot no. AST-1402; ALP kit catalog no. 89045, lot no. ALP-1398).

### Humoral immune response

To evaluate humoral immunity, four birds per replicate were wing-banded and injected intramuscularly with 0.1 mL of a 5 % suspension of sheep red blood cells (SRBC) in phosphate-buffered saline (PBS) at 10 d of age. Blood samples were collected from the wing vein at 17 d (for the primary response) and 21 d (for the secondary response) of age to determine antibody titers against SRBC. The antibody response was assessed using the hemagglutination assay described by Cheema et al. (2003) and results were expressed as log₂ of the reciprocal of the highest serum dilution showing visible agglutination.

### Statistical analysis and modeling

All data were analyzed at the pen level using JAMOVI (Version 2.7.5.0). For each response variable, a one-way ANOVA was applied with dietary Trp level as the fixed factor. The partial η² values with 95 % CIs were reported as measures of effect size. Assumptions of normality and homogeneity of variance were verified by Shapiro–Wilk and Levene tests. Orthogonal polynomial contrasts (linear, quadratic, and higher-order if significant) were performed to assess dose–response trends across the seven equally spaced Trp levels (0.21–0.27 %). All results were expressed as mean ± SEM, significance was declared at *P* < 0.05 was considered. In addition to ANOVA and polynomial trend tests, nutrient optima were estimated using a rational nonlinear regression model to accurately determine the inflection point of each response curve using the following equation:

$$Y=\;\frac{\left(a_{0}+\;a_{1}X+\;a_{2}X^{2}\right)}{\left(1+\;b_{1}X+\;b_{2}X^{2}\right)}$$

where Y is the dependent variable, X represents Trp concentration, and a₀–b₂ are model coefficients estimated by nonlinear regression. This model form accommodates curvilinear and asymptotic responses typical of biological data. Nonlinear regression was performed separately for each response variable using the Levenberg–Marquardt algorithm implemented in the `*nlsLM()*` function (package “*minpack.lm*”) in R project (version 4.5.1). Initial parameter values (a₀–b₂) were obtained from a second-order polynomial regression of the form *y* = β₀ + β₁*x* + β₂x². Parameter bounds were set between −10 and +10 for denominator terms to avoid singularities. Model summaries were extracted using the “*broom*” and “*dplyr*” packages.

Goodness of fit was assessed using the coefficient of determination (R²) and the root mean square error (RMSE), calculated as:

$$R^{2}=1-\left(\frac{\sum {\left(y_{i}-\;{\hat{y}}_{i}\right)}^{2}}{\sum {\left(y_{i}-\;\bar{y}\right)}^{2}}\right)$$

$$RMSE=\;\sqrt{\sum {\left(y_{i}-\;{\hat{y}}_{i}\right)}^{2}\;/\;\left(n-k\right)}$$

where yᵢ and ŷᵢ denote observed and predicted values, n is the number of observations, and k is the number of parameters.

## Results

### Growth performance

**Fig. 1:**
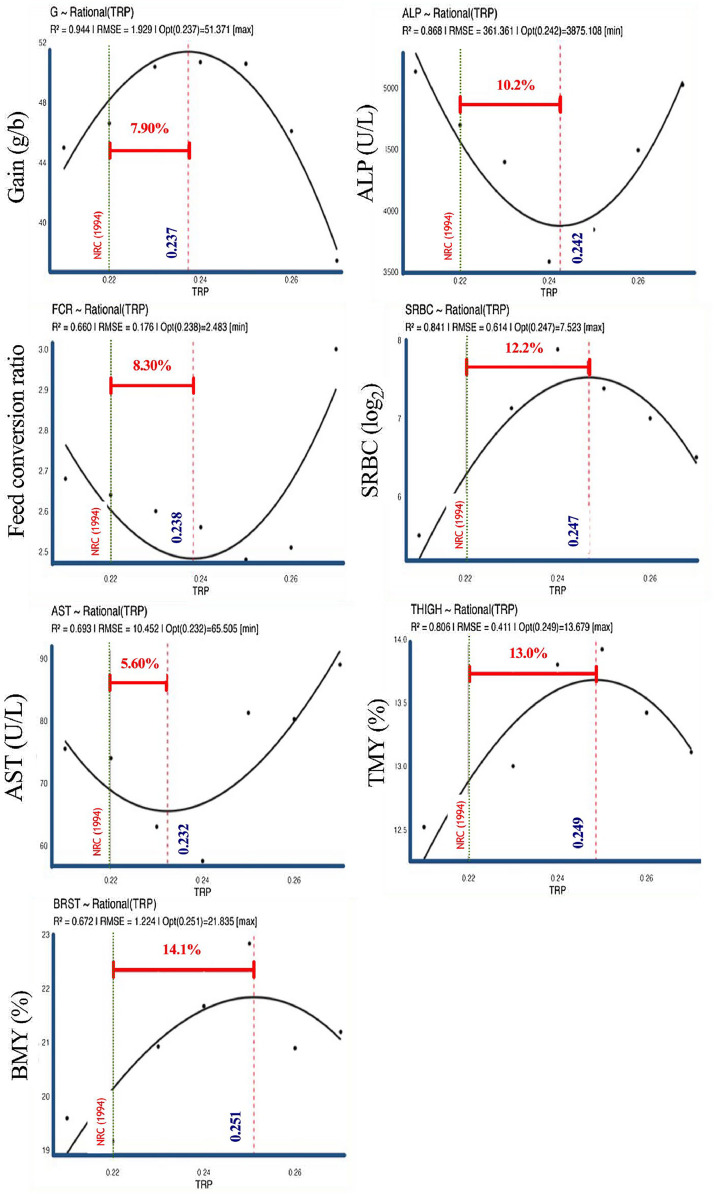
Modeling nonlinear responses of growing quails to increasing dietary tryptophan (TRP) using rational model regression. SRBC: sheep red blood cells, BMY: breast meat yield, TMY: thigh meat yield, AST: aspartate transaminase, ALP: alkaline phosphatase.

Dietary Trp supplementation significantly affected the growth performance of quails (Table 1, Table 2, Table 3). Gain increased linearly (*P* = 0.027) and quadratically (*P* < 0.001), showing a clear overall response (*P* = 0.011). The maximum G (50.7 g/bird) occurred at 0.24 % Trp, after which performance declined. FCR showed similar linear (*P* = 0.008) and quadratic (*P* = 0.006) trends, with the lowest value (2.48) at 0.25 % Trp, indicating peak feed efficiency. Feed intake was not significantly affected (*P* = 0.072). Large effect sizes (η²ₚ = 0.518 for gain; 0.504 for FCR) confirm the biological importance of Trp in growth response. According to the rational regression model, the optimal dietary Trp level was 0.237 % for G and 0.238 % for FCR (Fig. 1), representing 7.9 % and 8.3 % higher requirements than those recommended by the NRC (1994).

**Table 1:** Composition of basal diet.

Ingredient	Usage (%)
Wheat, Red W.	49.70
Corn Gluten Meal	19.33
Corn, Grain	15.51
Soybean Meal-44	7.91
Corn Starch	2.01
Limestone	1.49
Dical. Phos	1.09
L-Lysine HCl	0.89
NaHCO3	0.80
L-Arg	0.32
Mineral Premix^1^	0.25
Vitamin Premix^2^	0.25
DL-Methionine	0.20
L-Thr	0.13
L-Val	0.05
L-Ile	0.04
NaCl	0.03
Nutrient composition	
ME (kcal/kg)	2950
CP (%)	25.0
Methionine (%)	0.67
Lysine (%)	1.33
Threonine (%)	0.92
Tryptophan (%)	0.21
Ca (%)	0.80
P available (%)	0.30
Na (%)	0.26
K (%)	0.50
Cl (%)	0.24
DEB (mEq/kg)^3^	250

1 Mineral premix provided per kilogram of diet: Mn (from MnSO4·H2O), 65 mg; Zn (from ZnO), 55 mg; Fe (from FeSO4·7H2O), 50 mg; Cu (from CuSO4·5H2O), 8 mg; I [from Ca (IO3)2·H2O], 1.8 mg; Se, 0.30 mg; Co (from Co2O3), 0.20 mg; Mo, 0.16 mg.

2 Vitamin premix provided per kilogram of diet: vitamin A (from vitamin A acetate), 11,500 U; cholecalciferol, 2100 U; vitamin E (from dl-α-tocopheryl acetate), 22 U; vitamin B12, 0.60 mg; riboflavin, 4.4 mg; nicotinamide, 40 mg; calcium pantothenate, 35 mg; menadione (from menadione dimethyl-pyrimidinol), 1.50 mg; folic acid, 0.80 mg; thiamine, 3 mg; pyridoxine, 10 mg; biotin, 1 mg; choline chloride, 560 mg; ethoxyquin, 125 mg.

3 DEB: dietary electrolyte balance represents dietary Na + *K* – Cl in mEq/kg of diet.

**Table 2:** Analysis of variance of dietary tryptophan effects on weight gain, feed conversion ratio (FCR), breat meat yield (BMY), thigh meat yield (TMY), alkaline phosphatase (ALP), aspartate aminotransferase (AST), and antibody titer againt sheep red blood cell (SRBC) in growing Japanese quail.

Response	Tryptophan (%)							SEM	Probablity			η²p
	0.21	0.22	0.23	0.24	0.25	0.26	0.27		ANOVA	Linear	Quadratic	
Feed intake (g/b)	120	121	134	130	133	116	117	5.04	0.072	0.426	0.008	0.397
Gain (g/b)	44.9	46.6	50.4	50.7	50.6	46.1	37.4	2.88	0.011	0.027	< 0.001	0.518
FCR	2.68	2.64	2.6	2.56	2.48	2.51	3.00	0.14	0.014	0.008	0.006	0.504
BMY (%)	19.6	19.2	20.9	21.7	22.8	20.9	21.2	0.72	0.032	0.015	0.046	0.454
TMY (%)	12.5	12.7	13.0	13.8	13.9	13.4	13.1	0.22	0.009	0.013	0.028	0.526
ALP (U/L)	5132	4696	4396	3584	3845	4492	5023	213	< 0.001	0.268	< 0.001	0.674
AST (U/L)	75.5	74.0	63.0	57.5	81.3	80.3	89.0	2.88	< 0.001	< 0.001	< 0.001	0.803
SRBC (log_2_)	5.50	5.63	7.13	7.88	7.38	7.00	6.50	0.49	0.068	0.308	0.008	0.402

**Table 3:** Estimated parameters of the Rational model describing growth, carcass, and biochemical responses of growing quails to graded dietary tryptophan levels.

Response	Parameters					R^2^	RMSE	Optimal Trp	SE of estimate	95 % confidence intervals	
	a_0_	a_1_	a_2_	b_1_	b_2_					Lower	Upper
Gain	−557	5242	−11316	2.239	−10	0.944	1.929	0.237	0.002	0.239	0.247
FCR	22.65	−164	330	2.149	−10	0.660	0.176	0.238	0.001	0.254	0.256
BMY	−76.71	821	−1783	1.657	−10	0.672	1.224	0.251	0.005	0.236	0.258
TMY	−39.48	450	−997	1.661	−10	0.806	0.411	0.249	0.000	0.245	0.246
ALP	86615	−673345	1368576	2.355	−10	0.868	361	0.242	0.001	0.241	0.246
AST	4389	−36562	80753	10.00	10	0.693	10.45	0.232	0.002	0.233	0.241
SRBC	−98.94	879.207	−1824	2.175	−10	0.841	0.614	0.247	0.001	0.235	0.241

FCR: feed conversion ratio; BMY: breat meat yield; TMY: thigh meat yield; ALP: alkaline phosphatase; AST: aspartate aminotransferase; SRBC: sheep red blood cell.

### Carcass characteristics

BMY (*P* = 0.032) and TMY (*P* = 0.009) were both significantly influenced by dietary Trp, with pronounced linear (*P* = 0.015 and 0.013, respectively) and quadratic (*P* = 0.046 and 0.028, respectively) responses. The highest BMY (22.8 %) and TMY (13.9 %) were achieved at 0.25 % Trp, after which both traits slightly declined. These results indicate an optimal Trp concentration of approximately 0.24–0.25 % for maximizing carcass yield. Effect sizes were moderate to large (η²ₚ = 0.454 for BMY and 0.526 for TMY), highlighting the importance of dietary Trp in carcass muscle development. Model-based estimates indicated optimal Trp levels of 0.251 % for BMY and 0.249 % for TMY (Fig. 1), corresponding to 14.1 % and 13.0 % higher requirements than the NRC (1994) values.

### Hepatic enzymes

Serum enzyme activities were markedly affected by dietary Trp. ALP activity exhibited a highly significant overall response (*P* < 0.001), characterized by a strong quadratic pattern (*P* < 0.001). The lowest ALP concentration (3584 U/L) was recorded at 0.24 % Trp, with a large effect size (η²ₚ = 0.674). AST activity showed an even stronger response (*P* < 0.001 for overall, linear, and quadratic effects). The concentration decreased sharply to 57.5 U/L at 0.24 % Trp and increased thereafter, indicating a U-shaped response. The effect size for AST (η²ₚ = 0.803) was very large, suggesting high physiological sensitivity of hepatic enzymes to Trp status. The rational regression model estimated optimal Trp concentrations of 0.242 % for ALP and 0.232 % for AST (Fig. 1), which are 10.2 % and 5.6 % greater than the NRC (1994) recommendations.

### Immune response

Although the overall ANOVA for antibody titer against SRBC was not significant (*P* = 0.068), a significant quadratic effect (*P* = 0.008) was observed. The highest antibody titer (7.88 log₂) was obtained at 0.24 % dietary Trp, coinciding with the optimal levels for growth and enzymatic stability. The moderate effect size (η²ₚ = 0.402) indicates that dietary Trp may enhance immune responsiveness through improved metabolic and physiological conditions. Based on rational model analysis, the optimal dietary Trp level for maximum SRBC antibody response was 0.247 % (Fig. 1), representing a 12.2 % higher requirement than the NRC (1994) recommendation.

## Discussion

In the present study, dietary Trp elicited marked effects on growth performance, carcass traits, hepatic enzyme activities, and immune response in growing Japanese quails. Most performance and physiological traits responded non-linearly (quadratically) to increasing Trp, with optimum responses occurring around 0.24–0.25 % dietary Trp.

### Growth performance

The increase in body weight gain and improved FCR with increasing dietary Trp up to 0.24–0.25 % likely reflect enhanced protein deposition efficiency, reduced catabolic loss, and possible hormonal modulation. Trp serves as a precursor for serotonin and melatonin and participates in the kynurenine pathway involved in immune modulation and redox balance (Fouad et al., 2021). These mechanisms have been described mainly in mammals and chickens and should therefore be considered potential explanations in quails until further avian-specific evidence becomes available. One plausible mechanism is that Trp supplementation increases central serotonin (5-hydroxytryptamine) synthesis, which may influence satiety, feed intake regulation, and stress responses. In conditions of stress or marginal amino acid supply, limited Trp availability could reduce serotonin synthesis and impair neuroendocrine balance, potentially lowering feed efficiency. Melatonin, derived from serotonin, may also contribute to antioxidant regulation and nutrient absorption (Fouad et al., 2021). Previous studies in broilers have shown that Trp levels above the basal requirement can improve growth and FCR, particularly under stress or immune challenge (Mund et al., 2020). However, excessive Trp may cause amino acid imbalance—such as competition for transporters or increased nitrogen excretion—leading to the decline observed at higher inclusion levels. The relatively large partial eta squared values (*η*²ₚ = 0.52 for body weight gain; 0.504 for FCR) indicate substantial statistical effects but should be interpreted cautiously, as large effect sizes do not necessarily imply direct biological causation given the study’s sample limitations. Since feed intake was not significantly affected (*P* = 0.072), the improvements in gain and FCR most likely resulted from more efficient nutrient utilization—such as enhanced protein synthesis and reduced catabolism—rather than increased feed consumption. Overall, these findings suggest that dietary Trp levels of approximately 0.24–0.25 % support optimal growth efficiency in quails, although further mechanistic studies are needed to validate these physiological pathways in this species.

### Carcass characteristics

The observation that both BMY and TMY peaked at 0.25 % Trp with significant linear and quadratic responses suggests that muscle protein accretion in quails is highly responsive to Trp supply. Because muscle growth depends on amino acid availability and the balance between anabolic and catabolic signaling, adequate Trp may support more efficient muscle deposition by promoting protein synthesis and reducing catabolic losses (Emmert et al., 1997). Mechanistically, sufficient Trp could decrease the need for protein catabolism to supply Trp or its metabolites, thereby freeing more amino acids for muscle synthesis. Furthermore, Trp-derived metabolites may modulate immune status and oxidative stress; by potentially reducing inflammation or oxidative damage, more metabolic energy could be directed toward tissue growth (Linh et al., 2021). In poultry, carcass traits sometimes respond positively to supplemental Trp, particularly under stress or immune challenge, although the magnitude and direction of responses vary among species and depend on diet formulation (Linh et al., 2021). In quails, El-Kholy et al. (2024) reported that dietary Trp, with or without canthaxanthin, decreased hepatic enzyme activities (ALP and AST) and improved overall performance, but did not produce consistent effects on carcass traits. The observed effect sizes (η²ₚ = 0.45 for BMY, 0.53 for TMY) suggest dietary Trp significantly impacts muscle yield, highlighting its importance for carcass development. These findings are in agreement with those of Mehri et al. (2021), who showed that live weight, dressing percentage, BMY, and TMY all responded linearly or quadratically to Trp supplementation, demonstrating a strong dependence of carcass attributes on dietary Trp levels.

### Hepatic enzymes

One of the most striking findings of our study is the strong quadratic reduction in ALP and AST activities at 0.24 % Trp: ALP reached its minimum (3,584 U/L) and AST its lowest point (57.5 U/L), with significant responses and large effect sizes (η²ₚ = 0.674 for ALP, 0.803 for AST). These enzyme activities serve as biomarkers of hepatic (and perhaps systemic) tissue stress, cellular turnover, or membrane integrity. Lower values, as the optima, typically suggest better hepatic function, reduced cytotoxic leakage, or less metabolic stress. The mechanism by which dietary Trp suppresses ALP and AST activities may involve reduced oxidative stress, improved antioxidant defenses, and lower inflammatory signaling. Trp metabolites (via the kynurenine pathway) and downstream antioxidants (e.g., kynurenic acid) may mitigate reactive oxygen species and maintain membrane stability, thereby lowering enzyme leakage (El-Kholy et al., 2024; Ghazaghi et al., 2024). Under stress or toxin challenge, excess Trp has ameliorated enzyme elevations by supporting hepatic resilience in quail chicks (Ghazaghi et al., 2024). However, our data suggest that the 0.24 % Trp level provides a hepatic protective effect, beyond simply supplying substrate for growth. The fact that enzyme levels increased again at higher Trp levels indicates a tipping point where excess may become counterproductive, perhaps imposing metabolic detoxification costs or imbalance, in normal condition.

### Immune response

Although the overall ANOVA for SRBC antibody titer (log₂) was not statistically significant (*P* = 0.068), the presence of a significant quadratic component (*P* = 0.008) and a clear peak at 0.24 % Trp (7.88 log₂) suggests that humoral immune response is modulated by dietary Trp, albeit more subtly than growth or biochemical traits. The effect size (η²ₚ = 0.402) is moderate, indicating that Trp explains some portion of variation. Trp is well known to play immunoregulatory roles via multiple pathways. It is metabolized in part through the indoleamine 2,3-dioxygenase (IDO) pathway in immune tissues, which influences T-cell proliferation, tolerance, and immune homeostasis (Ghazaghi et al., 2024). In addition, serotonin and melatonin (derived from Trp) have modulatory effects on immune cells, promoting balance and reducing inflammatory stress (Fouad et al., 2021). Under suboptimal Trp, immune cell proliferation or antibody synthesis may be limited by substrate deficiency. Under excessive Trp, metabolic byproducts (e.g. kynurenine) may exert immunosuppressive effects. Thus, the observed quadratic response fits well with a “sweet spot” model. Some studies in poultry have found enhanced immune responses (e.g. increased Ig levels, improved lymphoid organ indices) with supplemental Trp, especially under stress conditions (Li et al., 2023). When interpreting these results together, a coherent mechanistic picture emerges that dietary Trp in the range of 0.24–0.25 % optimizes the balance between protein synthesis, metabolic stress, oxidative balance, and immune function. Below this level, Trp supply is limiting, reducing growth, increasing enzyme leakage, and blunting immune and muscle responses. Above this level, excess Trp may impose metabolic burden, generate more catabolic byproducts, lead to amino acid imbalance, or increase oxidative load, which is consistent with the slight downturns in performance and increase in enzyme activities at the highest levels.

### Statistical modeling

The use of quadratic regression (rational model) and reporting of η²ₚ in the present study offers a robust framework for identifying optimal dietary levels and assessing the proportion of variance explained. The large η²ₚ values for AST and ALP suggest that these biomarkers are highly responsive and thus useful as sensitive endpoints in nutrition research, perhaps even better than growth alone in some contexts. From a practical standpoint, feeding quail 0.24–0.25 % Trp would maximize growth and carcass yield while minimizing hepatic stress and likely supporting humoral immunity. In terms of formulation, one might start with a digestible amino acid matrix and adjust Trp accordingly. Additionally, because Trp interacts with other amino acids (e.g. competing for transport and catabolism), one should ensure balanced levels of lysine, methionine, and other limiting amino acids. Under challenging or stress-inducing conditions (e.g., heat stress or pathogenic exposure), dietary Trp levels exceeding basal maintenance requirements may confer protective benefits through enhanced antioxidant capacity, immunomodulatory effects, and mitigation of cellular enzyme leakage. Indeed, several studies have demonstrated improved physiological resilience and stress tolerance with supplemental Trp (Khanipour et al., 2019; Li et al., 2023). Nevertheless, excessive supplementation should be approached with caution, as surpassing the optimal threshold may negate these benefits and lead to metabolic inefficiency or amino acid imbalance. Importantly, the confidence intervals for all estimated parameters did not overlap with the NRC (1994) reference value, confirming that the Trp requirements identified in this study are statistically and biologically higher than the NRC (1994) recommendations. This finding is consistent with previous reports indicating that modern quail strains have greater amino acid needs due to their enhanced growth potential, lean tissue accretion, and metabolic activity (El-Kholy et al., 2024; Ghazaghi et al., 2024; Mehri et al., 2021). The NRC (1994) guidelines, which were primarily established from growth-based endpoints in older, slower-growing quail genotypes, therefore fall outside the confidence range of the current estimates. This emphasizes the necessity for revising and updating nutrient recommendations to encompass broader physiological indicators such as immune function, hepatic stability, and metabolic efficiency.

### Beyond NRC (1994) standards

In the present study, dietary Trp exerted significant and nonlinear effects on growth performance, carcass development, hepatic enzyme activity, and immune responses in growing Japanese quails. Most productive and physiological parameters exhibited quadratic responses to incremental Trp levels, with optimal values converging around 0.24–0.25 % dietary Trp. Body weight gain and FCR reached their optima near this range, while BMY and TMY also peaked at similar concentrations, indicating that Trp plays a pivotal role in muscle protein accretion and carcass quality. Likewise, serum liver enzymes (ALP and AST) attained their lowest, most favorable values, and antibody titers against SRBC were maximized near the same Trp range, underscoring the amino acid’s involvement in hepatic stability and immune modulation. These findings align with previous reports (Ghazaghi et al., 2024; Mehri et al., 2021) showing that the Trp requirements of modern Japanese quails exceed those proposed by the NRC (1994). The higher requirements observed, approximately 8–14 % above NRC (1994) values, can be attributed to several physiological and metabolic factors associated with contemporary poultry genotypes. Modern quails have been selected for accelerated growth, greater feed efficiency, and improved carcass yield, all of which are accompanied by enhanced rates of protein turnover and amino acid utilization (Emmert et al., 1997; Kidd et al., 2021). As a result, a larger proportion of dietary Trp may be directed toward supporting elevated protein synthesis and maintenance processes. Additionally, part of the ingested Trp is diverted from protein synthesis to non-protein metabolic pathways, including the serotonin, melatonin, and kynurenine routes. These metabolites are involved in neuroendocrine regulation, antioxidant defense, and immune modulation (Fouad et al., 2021; Linh et al., 2021), leading to increased physiological demand for Trp under intensive production or environmental stress (Ghazaghi et al., 2024; Khanipour et al., 2019). Furthermore, modern quail strains may experience greater oxidative and metabolic stress due to higher metabolic rates and dense rearing conditions, which further elevate the need for Trp as a precursor for stress-buffering compounds such as melatonin and kynurenic acid (El-Kholy et al., 2024; Li et al., 2023). Consequently, the elevated Trp requirements observed in the present study likely reflect the combined effects of enhanced growth potential, greater amino acid turnover, and increased diversion of Trp toward functional metabolic pathways beyond simple protein synthesis. Similar patterns have been documented in broiler chickens, where Trp supplementation above NRC (1994) levels improved growth rate, immune competence, and stress resilience (Fouad et al., 2021; Mund et al., 2020). Collectively, these insights suggest that modern nutrient requirement standards should move beyond purely performance-based endpoints to include indicators of immune function, oxidative balance, and metabolic efficiency. This broader perspective more accurately reflects the multifaceted role of Trp in sustaining both productivity and physiological robustness in high-performing quail genotypes.

In conclusion, the present study demonstrates that dietary Trp exerts substantial and nonlinear effects on growth, carcass characteristics, liver enzyme markers, and immune responses in growing quails. The convergence of optimal values around 0.24–0.25 % Trp indicates a reliable target range for achieving balanced performance and physiological stability. Because NRC (1994) recommendations were derived primarily from older, slower-growing strains, modern formulations should account for the expanded metabolic functions of Trp related to antioxidant protection, immune regulation, and neuromodulation. Future studies integrating metabolic profiling and stress-challenge assays will help refine these updated requirement estimates and enhance their applicability to diverse production systems.

## CRediT authorship contribution statement

**Mehran Mehri:** Writing – review & editing, Writing – original draft, Data curation, Conceptualization. **Mahmoud Ghazaghi:** Writing – review & editing, Software, Investigation, Funding acquisition. **Morteza Asghari-Moghadam:** Writing – review & editing, Supervision, Project administration, Data curation. **Amir Karamzadeh-Dehaghani:** Writing – review & editing, Validation, Project administration, Funding acquisition, Data curation. **Mohammad Rokouei:** Writing – review & editing, Validation, Resources, Investigation, Funding acquisition.

## Disclosures

The authors declare that they have no known competing financial interests or personal relationships that could have appeared to influence the work reported in this paper.
